# Effect of targeted estrogen delivery using glucagon-like peptide-1 on insulin secretion, insulin sensitivity and glucose homeostasis

**DOI:** 10.1038/srep10211

**Published:** 2015-05-13

**Authors:** Joseph P. Tiano, Chandra R. Tate, Bin S. Yang, Richard DiMarchi, Franck Mauvais-Jarvis

**Affiliations:** 1 Division of Endocrinology & Metabolism, Department of Medicine, Tulane University Health Sciences Center, School of Medicine, New Orleans, LA 70112; 2 Department of Medicine, Division of Endocrinology, Metabolism and Molecular Medicine, Northwestern University School of Medicine, Chicago, IL 60611; 3 Department of Chemistry, Indiana University, Bloomington, IN, 47405.

## Abstract

The female estrogen 17β-estradiol (E2) enhances pancreatic β-cell function via estrogen receptors (ERs). However, the risk of hormone dependent cancer precludes the use of general estrogen therapy as a chronic treatment for diabetes. To target E2 to β-cells without the undesirable effects of general estrogen therapy, we created fusion peptides combining active or inactive glucagon-like peptide-1 (GLP-1) and E2 in a single molecule (aGLP1-E2 and iGLP1-E2 respectively). By combining the activities of GLP-1 and E2, we envisioned synergistic insulinotropic activities of these molecules on β-cells. In cultured human islets and in C57BL/6 mice, both aGLP1 and aGLP1-E2 enhanced glucose-stimulated insulin secretion (GSIS) compared to vehicle and iGLP1-E2 without superior efficacy of aGLP1-E2 compared to GLP-1 alone. However, aGLP1-E2 decreased fasting and fed blood glucose to a greater extent than aGLP1 and iGLP1-E2 alone. Further, aGLP1-E2 exhibited improved insulin sensitivity compared to aGLP1 and iGLP1-E2 alone (HOMA-IR and insulin tolerance test). In conclusion, targeted estrogen delivery to non-diabetic islets in the presence of GLP-1 does not enhance GSIS. However, combining GLP-1 to estrogen delivers additional efficacy relative to GLP-1 alone on insulin sensitivity and glucose homeostasis in non-diabetic mice.

Protecting the functional mass of insulin-producing β-cells is a major therapeutic challenge in patients with type 2 diabetes (T2D). The gonadal hormone 17β-estradiol (E2) favours reproductive, bone, cardiovascular and neuronal physiology via activation of estrogen receptors (ERs)^1^. In pre-clinical models, we and others have shown that enhancing ER actions in β-cells protects survival and function in the face of multiple diabetic insults^2^,^3^,^4^,^5^,^6^,^7^,^8^,^9^,^10^. This is substantiated by two large, randomized placebo-controlled trials, the Women’s Health Initiative study^11^ and the Heart and Estrogen/Progestin Replacement study^12^ that found a significant reduction in the incidence of diabetes in women assigned to menopausal estrogen therapy, thus providing evidence for the antidiabetic action of estrogen in women. One of the major challenges of general estrogen therapy in diabetes, however, lies in the risk of hormone-dependent cancer if ERs are activated in breast and endometrial tissues^13^.

One of the most effective therapies for T2D involves activation of the receptor for the gut-derived hormone glucagon-like-peptide-1 (GLP-1). The actions of GLP-1 include incretin and satiety effects that are mediated via GLP-1 receptors (GLP-1R) in pancreatic islets and metabolic control centers in the hypothalamus^14^,^15^. We explored the preferential targeting of E2 to the islet β-cells through the use of GLP-1-based fusion peptides. By marrying insulinotropic activities of GLP-1 and E2, we envisioned a synergism on islet β-cells that express both ER and GLP-1 receptors (GLP-1R), while improving the therapeutic index of estrogen. Thus, conjugates with active GLP-1 made resistant to dipeptidyl peptidase-4 (DPP-4) and stably linked E2 were synthesized^16^. Such conjugates avoid E2 release in circulation and maximize E2 release in target cells. In diet-induced obese mice, we validated the synergistic anti-obesity ability of such peptide conjugates above those of single agonists, without inducing the adverse effects of systemic estrogen action^16^. In addition, in the New Zealand obese (NZO) mice, in contrast to GLP-1, the GLP-1 and E2 conjugate significantly decreased food intake and prevented obesity, thus preventing T2D^17^. However, the synergistic effect of GLP-1 and E2 on glucose homeostasis, independently from their synergism on fat mass has not been investigated. Here, using wild type mice and human islets, we tested the synergistic activity of such peptide conjugates on glucose-stimulated insulin secretion (GSIS), insulin sensitivity and glucose homeostasis.

## Results

We used a GLP-1 molecule covalently attached to E2 (aGLP1-E2). *In vitro* characterization and pharmacokinetics of this GLP-1–estrogen conjugate have been previously described^16^.

**Figure 1:**
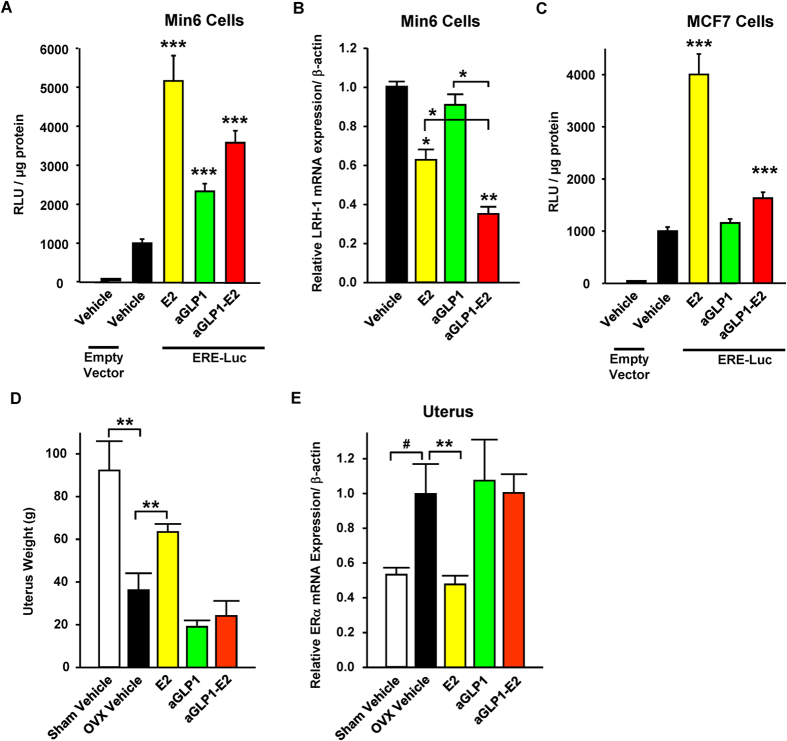
GLP-1 efficiently targets E2 to β-cells. (**A**) Relative luciferase activity normalized to protein levels in Min6 cells transfected with an ERE-Luc expression plasmid and treated with E2 (10 nM), aGLP1 (10 nM) or aGLP1-E2 (10nM). N = 6 from 2 independent experiments. (**B**) LRH-1 mRNA expression normalized to β-actin in Min6 cells treated with E2 (10 nM), aGLP1 (10 nM), or aGLP1-E2 (10 nM). N = 3 from 1 experiment. (**C**) Relative luciferase activity normalized to protein levels in MCF7 human breast cancer cells transfected with an ERE-Luc expression plasmid and treated with E2 (10 nM), aGLP1 (10 nM), or aGLP1-E2 (10 nM). N = 9 from 3 independent experiments (**D**) Uterus weight in female sham operated and ovariectomized (OVX) mice following two days of treatment with E2 (4 μg/mouse), aGLP1 (120 μg/kg) or aGLP1-E2 (120 μg/kg). (**E**) ERα mRNA expression normalized to β-actin in uterus from mice in part D. N = 5-12 mice per group. Results represent the mean ± SEM. *≤0.05, **≤0.01, ***≤0.001 vs. Vehicle when not indicated.

In order to investigate the functional targeting of β-cells by the aGLP1-E2 conjugate, we used Min6 insulin-secreting cells transfected with a luciferase reporter construct containing an estrogen response element (ERE-Luc)^7^. In these cells, E2 and aGLP1-E2 exhibited a marked transcriptional activity (5-fold and >3.5-fold, respectively; Fig. 1A). LRH-1 is a target gene of ERα in β-cells^18^. In Min6 cells E2 treatment suppressed LRH-1 mRNA but GLP-1 did not (Fig. 1B). However, treatment with aGLP1-E2 suppressed the expression of LRH-1 mRNA. Together, these results demonstrate the functional targeting of β-cells by E2 using the aGLP1-E2 conjugate (Fig. 1B). In contrast, in MCF7 human breast cancer cells, transfected with the same ERE-Luc reporter construct, the aGLP1-E2 compound showed weak transcriptional activity (<1.5-fold) compared to E2 (4-fold), further confirming our *in vivo* finding that aGLP1-E2 does not stimulate breast ER activity (Fig. 1C). Further, in the uterotrophic model of ovariectomized (OVX) mice we confirm that, unlike E2, aGLP1-E2 does not stimulate uterine growth (Fig. 1D). In the uterus, E2 suppresses the expression of ERα, which represents a molecular control of E2 action in this tissue *in vivo*^19^. Unlike E2, aGLP1-E2 did not suppress uterine expression of ERα mRNA, demonstrating the absence of activation of ERα in this tissue (Fig. 1E). In order to target estrogen to islets and establish the efficacy of estrogen divorced from GLP-1 activity, we also synthesized an inactive GLP-1 peptide stably linked to E2 (iGLP1-E2). This later conjugate binds GLP-1R but is incapable of activating GLP-1R signaling^16^.

**Figure 2:**
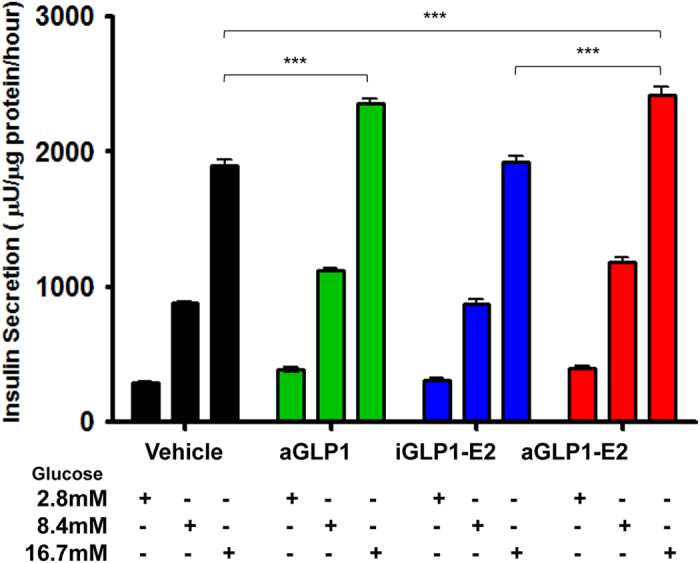
Effect of targeted estrogen delivery on GSIS in human islets. Static insulin secretion in human islets treated with aGLP1 (10 nM), iGLP1-E2 (10 nM) or aGLP1-E2 (10 nM) at the indicated glucose concentrations. Insulin secretion was normalized to protein content. Results represent the mean ± SEM. *≤0.05, **≤0.01, ***≤0.001. Islets came from one donor and each condition was performed in 6 biological replicates.

We assessed glucose-stimulated insulin secretion (GSIS) in static incubation in male human islets. Treatment with both the aGLP1 and aGLP1-E2 enhanced GSIS at 8.4 mM and 16.7 mM glucose, compared to vehicle and to the iGLP1-E2 (Fig. 2). However, we observed no additional enhancement of GSIS with the aGLP1-E2 compound compared to the aGLP1 alone (Fig. 2). In addition, we observed no stimulation of GSIS with iGLP1-E2 compared to vehicle (Fig. 2).

**Figure 3:**
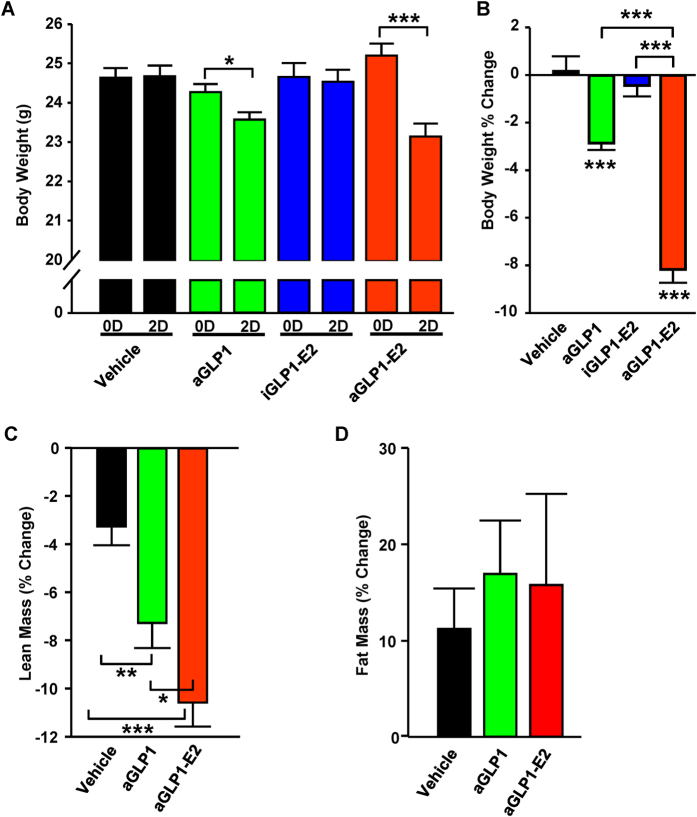
Effect of targeted estrogen delivery on body composition in WT mice. (**A**) Body weight in male WT mice following 48 hours treatment with aGLP1 (120 μg/kg), iGLP1-E2 (120 μg/kg) or aGLP1-E2 (120 μg/kg). (**B**) Percent change in fat mass and lean mass in mice from (**A**) N = 10 mice per group. (**C** and **D**) Percent change in (**C**) lean mass and (**D**) fat mass in a separate group of male mice treated the same. N = 8-12 mice per group Results represent the mean ± SEM * vs. Vehicle when not indicated. *≤0.05, **≤0.01, ***≤0.001.

We studied the effect of aGLP1, aGLP1-E2 or iGLP1-E2 *in vivo* during a 48 hour treatment period in wild type C57BL/6 mice. Treatment with aGLP1 significantly lowered body weight compared to vehicle (Fig. 3A,B) which was mostly due to a reduction in lean mass (Fig. 3C). Furthermore, as in the case of diet-induced obese mice^16^, treatment with aGLP1-E2 reduced body weight to an extent even greater than aGLP1 alone (Fig. 3A,B). Treatment with iGLP1-E2 showed no effect on body weight (Fig. 3A,B).

**Figure 4:**
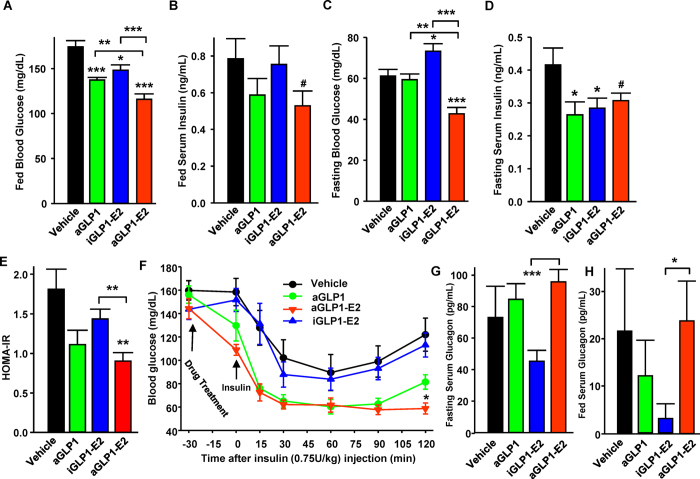
Effect of targeted estrogen delivery on glucose and insulin homeostasis in WT mice. (**A**) Fed blood glucose and (**B**) fed serum insulin in male WT mice treated with aGLP1 (120 μg/kg), iGLP1-E2 (120 μg/kg) or aGLP1-E2 (120 μg/kg) for 48 hours. (**C**) Fasting blood glucose and (**D**) fasting serum insulin in the same mice. (**E**) HOMA-IR calculated from C and D. (**F**) IP-ITT (0.75 U/kg) in male WT mice treated as above. (**G**) Fasting serum glucagon and (**H**) fed serum glucagon in male WT mice from A and B. N = 10 mice per group. Results represent the mean ± SEM. * vs. Vehicle when not indicated. *≤0.05, **≤0.01, ***≤0.001.

We next assessed the efficacy of these compounds on glucose homeostasis in the same mice. Treatment with aGLP1 produced a decrease in fed blood glucose as well as fasting and fed insulin levels, suggesting improved insulin sensitivity (Fig. 4A–D). The iGLP1-E2 conjugate decreased fed blood glucose and fasting serum insulin suggesting a minor improvement in glucose homeostasis (Fig. 4A,D). Importantly, treatment with aGLP1-E2 decreased fasting and fed blood glucose to an extent greater than aGLP1 alone (Fig. 4A,C). Surprisingly, iGLP1-E2 slightly increased fasting glucose while decreasing fasting insulin. The aGLP1-E2 did not significantly alter fasting or fed insulin levels compared to aGLP1 or iGLP1-E2 (Fig. 4B,D). We used HOMA-IR as a surrogate index of insulin sensitivity^20^. Neither aGLP1 nor iGLP1-E2 alone improved HOMA-IR (Fig. 4E). In contrast, aGLP1-E2 produced significant improvement in HOMA-IR compared to vehicle and iGLP1-E2, demonstrating improved insulin sensitivity (Fig. 4E). Further, during intraperitoneal (IP) insulin tolerance testing (ITT), aGLP1-E2 produced a more sustained enhancement of the hypoglycemic effect of insulin compared to vehicle or iGLP1-E2 (Fig. 4F), confirming results obtained in HOMA-IR (Fig. 4E). Treatment with the different compounds produced no significant effect on serum glucagon compared to vehicle, although both fasting and fed serum glucagon were significantly lower in mice treated with iGLP1-E2 compared to aGLP1-E2 (Fig. 4G,H).

**Figure 5:**
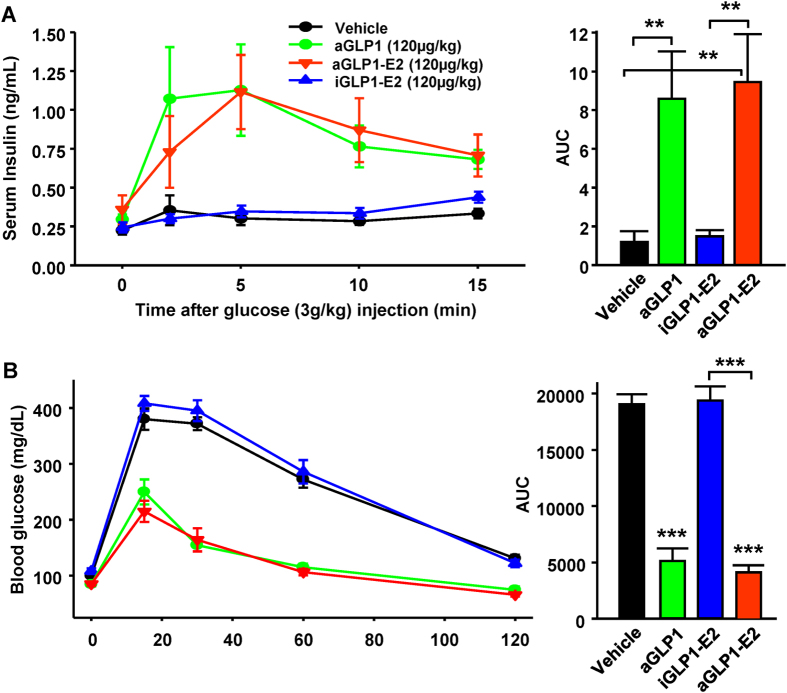
Effect of targeted estrogen delivery on GSIS and GTT in WT mice. (**A**) IP-GSIS with serum insulin and area under the curve (AUC) in male WT mice treated as above and injected subcutaneously with glucose (3.0 g/kg). (B) IP-GTT (2.0 g/kg) and AUC in male WT mice treated as above. N = 10 mice per group. Results represent the mean ± SEM. * vs. Vehicle when not indicated. *≤0.05, **≤0.01, ***≤0.001.

We assessed GSIS during an IP glucose load. Treatment with aGLP1 and aGLP1-E2 produced a robust and similar increase in acute insulin secretion compared to vehicle treated mice (Fig. 5A). Treatment with iGLP1-E2 did not induce insulin secretion over vehicle treatment (Fig. 5A). Accordingly, both aGLP1 and aGLP1-E2 but not iGLP1-E2 displayed improved glucose tolerance compared to vehicle treated mice during a glucose tolerance test (GTT) (Fig. 5B). The suppression in serum glucagon 15 minutes following a glucose challenge was similar among all groups (Suppl. Fig. 1).

## Discussion

Since we previously showed enhanced sex-independent efficacy of aGLP1-E2 over either E2 or aGLP1 alone to improve diet-induced obesity^16^, we used male mice in the present study to avoid the confounding effect of circulating estrogens in females. The fusion peptide combining GLP-1 and estrogen delivers an additional efficacy relative to the parent GLP-1 peptide on body weight suppression, which is likely due to the reduction in food intake already described^16^. GLP-1 exhibits incretin actions via the GLP-1R in β-cells^15^, and estrogen increases the biosynthesis and secretion of insulin via at least three ERs^2^,^9^,^10^,^21^,^22^. Therefore, we reasoned that the combination of both molecules in a single compound would not only target E2 to the islets but also provide synergistic insulintropic actions between E2 and GLP-1. Delivery of estrogen to β-cells using GLP-1 was successful, as demonstrated by the increased estrogen signaling in Min6 cells. However, targeted estrogen delivery to islets using GLP-1 did not enhance the incretin action of GLP-1 in cultured islets or *in vivo*. In addition, targeted estrogen delivery to islets did not enhance GSIS when GLP-1 was used as a dead vehicle. This suggests that GLP-1 and E2 do not exhibit synergistic insulinotropic actions in normal islets. This is consistent with a recent study showing that aGLP1-E2 protects Male New Zealand obese from β-cell failure via an extra islet effect involving suppression of hyperphagia and body weight^17^.

In contrast, the marriage of GLP-1 to estrogen delivers an additional efficacy relative to the parent GLP-1 peptide on blood glucose levels in fasting and fed conditions in wild type mice. The combination of GLP-1 to estrogen could improve insulin sensitivity over either of the individual hormones alone since insulin secretion is unchanged. Still, the HOMA-IR and the ITT do not show that aGLP1-E2 has provided a significant improvement in insulin sensitivity over aGLP1 alone. Further studies are needed to assess insulin sensitivity using more sensitive methods. This glucose-lowering effect of aGLP1-E2 over GLP-1 is independent from the anti-obesity effect already described^16^ since in the present study the aGLP1-E2 conjugate did not decrease fat mass. Likewise, the improvement in glucose homeostasis of aGLP1-E2 over either GLP-1 or iGLP-E2 alone is independent from glucagon suppression since we observed no effect of either molecule on serum glucagon. The synergism between GLP-1 and estrogen in enhancing glucose homeostasis could therefore result from several mechanisms in the periphery and the brain. First, we observed that treatment with the aGLP1-E2 conjugate induces a dramatic decrease in fasting glucose compared to either GLP-1 or iGLP-E2 suggesting an enhanced suppression of hepatic glucose production. There is a large body of evidence demonstrating that treatment with either GLP-1 or estrogen receptor agonists suppress fatty liver^23^,^24^,^25^ and improve hepatic insulin sensitivity^24^,^25^,^26^,^27^,^28^. Therefore, GLP-1 and estrogen may synergize to suppress hepatic glucose production in C57BL/6 mice, a model prone to metabolic diseases. Alternatively, GLP-1 and estrogen have documented effects in the CNS. For example, activation of central GLP-1R enhances peripheral insulin sensitivity in mice^29^. Activation of ERs in the hypothalamus also promotes energy homeostasis^13^ which may synergize with GLP-1R in the same neurons to enhance peripheral insulin sensitivity. Additional studies are necessary to address these issues.

In conclusion, targeted E2 delivery, using GLP-1 in a single molecule provides additional efficacy compared to either hormone alone on glucose homeostasis in normal male mice.

## Methods

### Cell and islet studies

Min6 β-cells were cultured in DMEM media (glucose 4.54 g/L) (Gibco) supplemented with 10% FBS (Gemini Bio-Products) or phenol-red free DMEM media (Sigma-Aldrich) with 10% charcoal-stripped FBS prior to experimentation. Human islets were obtained from the Integrated Islet Distribution Program (IIDP) and cultured as above. Donor Characteristics: Male, 50 year-old, BMI: 28.

### Drug treatments

The drugs used in culture studies were active glucagon-like peptide-1 (aGLP1, 10^−8^ M), active GLP-1 covalently fused to E2 (aGLP1-E2, 10^−8^ M) and inactive GLP-1 covalently fused to E2 (iGLP1-E2, 10^−8^M). The synthesis of GLP-1–estrogen conjugates has been previously described^16^. For *in vivo* studies, mice were treated with a once daily IP injection of aGLP1 (120 μg/kg), iGLP1-E2 (120μg/kg) or aGLP1-E2 (120 μg/kg) for a duration 48 hours. For each compound, the *in vivo* dose of 120 μg/kg is equivalent to the *in vitro* dose of 10^−8^ M^16^.

### Mice

C57BL/6 male mice were housed with free access to food and water with a 14:10 light:dark cycle. Experiments were approved by Northwestern University Animal Care and Use Committee and conducted in accordance with the National Institutes of Health guidelines.

### Insulin secretion in static incubation

Human islets were hand-picked and transferred to 24-well plate (10IEQ/well) containing Krebs Ringer Bicarbonate (130 mM NaCl, 3.6 mM HCl, 0.5 mM NaH_2_PO_4_, 0.5 mM MgSO^4^, CaCl) supplemented with HEPES (2.38 mg/ml) and BSA (1.0 mg/ml), with 0.1% Fatty Acid Free BSA (Sigma) and allowed to attach, overnight. Islets were pre-incubated 30 min in KRB,1% BSA with 2.8 mM Glucose followed by incubation in the presence of glucose (2.8, 8.35, 16.7 mM) and corresponding drug treatment–for 1 hour. Insulin was extracted by acid ethanol. Samples were stored at −20 °C until insulin quantification by ELISA (Millipore).

### Body composition

In vivo body composition analysis of lean mass and fat mass was performed on immobilized mice using quantitative magnetic resonance (Minispec LF90II, Bruker Optics, Woodlands, TX).

### Metabolic studies

In mice, blood glucose, serum insulin and glucagon were assessed using a glucose monitor (OneTouch Ultra; Lifescan), insulin and glucagon ELISA (Linco Research). The intraperitoneal glucose tolerance test (IP-GTT, 2.0 g/kg) and the IP-GSIS (3.0 g/kg) were performed following an 18 h fast. Mice were injected IP with the drugs 30 minutes prior to glucose challenge. An IP insulin tolerance test (ITT) was performed following a 6 h fast (insulin 0.75 U/kg). Blood glucose and serum insulin were measured as described above. HOMA-IR was calculated as fasting insulin (mIU/L) x glucose (mg/dL) / 405. Fat mass and lean mass were assessed using DEXA.

### Quantitative real time-PCR

Total RNA was extracted from cells or tissue in TRIzol Reagent (Invitrogen) and 1.0 μg of RNA was reverse transcribed using iScript cDNA Synthesis Kit (Bio-Rad) with random hexamers (iCycler, Bio-Rad). mRNA expression of target genes was quantified using iQ SYBR Green Supermix (Bio-Rad).

### Luciferase assay

Min6 cells and MCF7 cells were transfected for 6 h with 2 μL of Lipofectamine 2000 (Invitrogen) and 0.8 μg of ERE-Luc in 0.5 ml phenol red free RPMI 1640 media and treated with the respective drugs for 24 h. Luciferase assay (Promega) was performed according to manufactures instructions.

### Statistical analysis

All results are presented as mean ± SEM unless otherwise stated. All data was analyzed using the unpaired two-tailed Student’s t test. A value of p < 0.05 was considered statistically significant.

## Author Contributions

J.P.T. and C.R.T. performed experiments, data acquisition and analysis and drafted the manuscript. B.S.Y. and R.D. synthesized and provided reagents. F.M.J. designed the study, interpreted the results, wrote and reviewed the manuscript.

## Additional Information

**How to cite this article**: Tiano, J. P. *et al.* Effect of targeted estrogen delivery using glucagon-like peptide-1 on insulin secretion, insulin sensitivity and glucose homeostasis. *Sci. Rep.* **5,** 10211; doi: 10.1038/srep10211 (2015).

## Supplementary Material

Supplementary Information
