# Supplementary material for: Effect of targeted estrogen delivery using glucagon-like peptide-1 on insulin secretion, insulin sensitivity and glucose homeostasis
Source: Sci Rep. 2015 May 13;5:10211. doi: 10.1038/srep10211 (PMC4429560; doi:10.1038/srep10211)
Supplement: Supplementary Information [file srep10211-s1.doc]

**Supplementary information**

**Effect of targeted estrogen delivery using glucagon-like peptide-1 on insulin secretion, insulin sensitivity and glucose homeostasis**

Joseph P. Tiano, Chandra R. Tate, Bin S. Yang, Richard DiMarchi, Franck Mauvais-Jarvis

**Supplementary Figure 1.** **Effect of targeted estrogen delivery on glucagon in WT mice.** Serum glucagon measured at the 15 minute time point in male WT mice (From Figure 4H) treated with aGLP1 (120µg/kg), iGLP1-E2 (120µg/kg) or aGLP1-E2 (120µg/kg) and injected subcutaneously with glucose (2.0g/kg). n=8-10 mice per group. Results represent the mean ± SEM. * vs. Vehicle when not indicated. *≤0.05, **≤0.01, ***≤0.001.
